# Identification of Mycobacterium tuberculosis isolated from culture-negative pulmonary and extra-pulmonary samples in cases of suspected tuberculosis

**DOI:** 10.3205/dgkh000325

**Published:** 2019-08-05

**Authors:** Saeed Harirzadeh, Mohammad Javad Kazemi, Sajad Babakhani

**Affiliations:** 1 Medical Biotechnology Research Center, Ashkezar Branch, Islamic Azad University, Ashkezar, Yazd, Iran; 2 Department of Microbiology, North Tehran Branch, Islamic Azad University, Tehran, Iran

**Keywords:** latent tuberculosis, Mycobacterium tuberculosis, PCR, real-time PCR, rrnA-PCL1

## Abstract

**Introduction:** *Mycobacterium tuberculosis* is the cause of pulmonary and extra-pulmonary tuberculosis. Latent tuberculosis is asymptomatic and the results of mycobacterial culture in this case are negative. The purpose of this study was to determine the prevalence of *M. tuberculosis* in negative cultures of pulmonary and extra-pulmonary samples from suspected tuberculosis patients in Yazd city.

**Methods:** Specimens were collected from 44 patients with suspected pulmonary and extra-pulmonary tuberculosis. The culture result of all samples was negative and DNA was extracted from culture-negative samples. Then the IS*6110* sequence of *M. tuberculosis* was determined by PCR, and the presence of 16S rRNA and *rrn*A-PCL1 was checked by real-time PCR. Data were analyzed using SPSS software.

**Results:** Of the 44 pulmonary and extra-pulmonary samples, *M. tuberculosis* DNA was detected in 12 cases (27%) by PCR. Of the 12 positive cases of *M. tuberculosis*, 9 were detected in lung samples and 3 in extra-pulmonary samples. For *rrn*A-PCL1 and 16S rRNA genes, 10 samples (23%) were confirmed positive by real-time PCR. The frequency distributions of *M. tuberculosis* according to PCR and real-time PCR were not significantly different (p=0.403).

**Conclusion:** Latent tuberculosis is characterized by the absence of clinical symptoms and the absence of bacilli in the culture. Regardless of the statistical analysis of results, PCR more effectively detects *M. tuberculosis* than does real-time PCR. According to the results, detection of *M. tuberculosis* in pulmonary and extra-pulmonary samples with culture-negative by PCR is reliable.

## Zusammenfassung

**Einleitung:** *Mycobacterium tuberculosis* ist die Ursache der pulmonalen und extra-pulmonalen Tuberkulose. Die latente Tuberkulose verläuft asymptomatisch und die Ergebnisse der mykobakteriellen Kultur sind unter diesen Bedingungen negativ. In der Studie sollte die Prävalenz von *M. tuberculosis* bei negativem Kulturergebnis pulmonaler und extra-pulmonaler Proben von Patienten mit Verdacht auf Tuberkulose in der Stadt Yazd bestimmt werden.

**Methoden:** Bei 44 Patienten mit Verdacht auf pulmonale und extra-pulmonale Tuberkulose wurden Proben entnommen. Das Kulturergebnis aller Stichproben war negativ und DNA wurde aus Kultur-negativen Stichproben extrahiert. Dann wurde die IS*6110*-Sequenz von *M. tuberculosis* durch PCR nachgewiesen und das Vorhandensein von 16S rRNA und *rrn*A-PCL1 durch real-time PCR überprüft. Die Daten wurden mit SPSS-Software analysiert.

**Ergebnisse:** Von 44 pulmonalen und extra-pulmonalen Proben wurde *M. tuberculosis*-DNA in 12 Fällen (27%) mittels PCR nachgewiesen. Von den 12 positiven Fällen wurden 9 in pulmonalen und 3 in extra-pulmonalem Material nachgewiesen. Für *rrn*A-PCL1- und 16S rRNA-Gene wurden 10 Proben (23%) durch real-time PCR positiv bestätigt. Der p-Wert für die Häufigkeitsverteilung von *M. tuberculosis* durch PCR und real-time PCR wurde mit 0,403 bestimmt.

**Schlussfolgerung:** Die latente Tuberkulose ist gekennzeichnet durch Fehlen klinischer Symptome und Abwesenheit von Bakterien in der Kultur. Unabhängig von der statistischen Analyse der Ergebnisse ist die PCR zum Nachweis von *M. tuberculosis* effektiver als real-time PCR und zum Nachweis von *M. tuberculosis* in pulmonalen und extra-pulmonalen Proben bei negativer Kultur geeignet.

## Introduction

Tuberculosis (TB) is one of the oldest and deadliest diseases in the world, and is caused by *Mycobacterium tuberculosis*. In some areas, e.g., some parts of Africa, TB is caused by *Mycobacterium africanum *[1]. The lack of an effective vaccine and antibiotic resistance to *M. tuberculosis* has led to an increase in the cost of treatment for patients with TB [1], [2].

Antibiotic resistance in *M. tuberculosis* is due to mutations in the *rpoB* (rifampicin), *katG* and *inhA*-promoter (isoniazid), *rpsL* (streptomycin) and *embB* (ethambutol) genes [3]. The genome of *M. tuberculosis* is about 4,300 kb [4], [5]. For example, the genome of *M. tuberculosis* that was sequenced by the Sanger and Illumina methods is 4,385 kb long [4]. The complete genome length of *M. tuberculosis*, CCDC 5079 and CCDC 5080 strains, is 4,399 kb and 4,406 kb, respectively [5].

Individuals with active TB are major reservoirs of *M. tuberculosis*, which can infect healthy people. Active TB has clinical manifestations, and the culture of the patient’s sputum is positive. Latent TB is symptom-free and does not cause any abnormalities in the patient. Due to the possibility of activation of latent TB, diagnosis and treatment of the disease is very important [6]. Latent TB is activated in patients with a deficient immune system (e.g., due to AIDS). The prevalence of active TB is significantly lower than that of latent TB. Therefore, the study of the global prevalence of latent TB is of particular importance [7].

TB manifests clinically as two types: pulmonary TB (PTB) and extra-pulmonary TB (EPTB). EPTB involves organs such as lymph nodes, genital tract, skin, and joints. In some people with EPTB, sputum culture may be positive, while these people have normal lung function [8].

PCR is a specific and rapid method for detecting EPTB and PTB [8]. In general, molecular methods are well suited for the detection of *M. tuberculosis*. The Xpert MTB/RIF method also increases the sensitivity for TB diagnosis [9]. The sensitivity and specificity of the real-time PCR method (RT-PCR) for the detection of PTB and EPTB are 80% and 100%, respectively. Molecular methods for the detection of *M. tuberculosis* in clinical specimens with negative culture are also very promising [10]. Therefore, the aim of this study was to evaluate the frequency of *M. tuberculosis* with metabolic activity in culture-negative clinical samples obtained from suspected TB patients.

## Methods

### Studied population

This study was conducted on samples obtained from 44 suspected TB patients. These people were referred to Shahid Sadoughi hospital in Yazd during 2016, because they had experienced symptoms of TB disease. The ethics committee of Shahid Sadoughi University of Medical Sciences and Shahid Sadoughi hospital approved this research.

### Sample collection 

In total, 24 pulmonary samples (8 sputum and 16 bronchial) and 20 extra-pulmonary samples (11 pleura, 2 synovia and 7 liquids) were collected. The samples were culture-negative in MGIT (BACT^TM^) liquid culture medium and Lowenstein-Jensen solid culture medium (Merck). Cultures with no growth in liquid medium after 45 days at 37°C were considered negative. Cultures with no growth in solid medium after 56 days were also considered negative. Positive cultures were identified by acid-fast fluorescent stain. The identification of *M. tuberculosis* was investigated in negative cultures.

### DNA and RNA extraction and analysis

To extract the DNA and RNA of *M. tuberculosis*, High Pure Nucleic Acid Kit (Roche Diagnostics, Germany) was used for all pulmonary and extra-pulmonary specimens. Tubes containing nucleic acid for PCR and RT-PCR reactions were stored at 4°C.

To evaluate the DNA of *M. tuberculosis* in pulmonary and extra-pulmonary specimens, IS1 and IS2 primers were used to identify the IS*6110* gene (Table 1 (Tab. 1)). The PCR reaction reagents included 5 µl reaction buffer, 1 µl taq polymerase enzyme, 1 µl dNTPs, 1 µl MgCl2, 0.5 µl forward primer, 0.5 µl reverse primer, 6 µl DNA sample and 5 µl distilled water. The cycling conditions were as follows: 40 cycles of denaturation at 94°C for 45 sec, annealing at 55°C for 45 sec and extension at 72°C for 10 sec. The PCR product was evaluated by agarose gel electrophoresis (1%–1.5%). In this method, Ladder (Fermentas, 50–100 bp), 5 µl of each sample and 1 µl of loading buffer were used. 

RT-PCR reaction was used to detect *rrn*A-PCL1 and 16S rRNA genes. The primers used in RT-PCR are indicated in Table 1 (Tab. 1). The reaction mixture consisted of 12 µL Master Mix (reaction buffer and SYBR Green), 0.5 µl forward primer, 0.5 µL reverse primer, 1 µl MgCl2, 1 µl dNTPs and 5 µL DNA sample. To verify the accuracy of RT-PCR, a lymphatic tissue sample from a person infected with *M. tuberculosis* and the plasma of a healthy person were used as positive and negative controls, respectively.

Initial denaturation for the RT-PCR was performed at 95°C for 10 min. The cycling conditions for 40 cycles were as follows: denaturation at 95°C for 15 sec, annealing at 55°C for 30 sec and extension at 72°C for 15 sec. Samples with a cycle threshold (CT) under 40 were considered positive. The results were analyzed by SPSS software. p=<0.05 was considered statistically significant.

## Results

Among 44 suspected TB patients, 24 were male and 20 female. Demographic data on the study subjects are shown in Table 2 (Tab. 2).

Of the 44 pulmonary and extra-pulmonary samples, PCR showed that 12 samples (27%) contained *M. tuberculosis* DNA. Of the 12 positive TB samples, 9 were pulmonary specimens and 3 were extra-pulmonary specimens (Table 3 (Tab. 3)).

**Figure 1:**
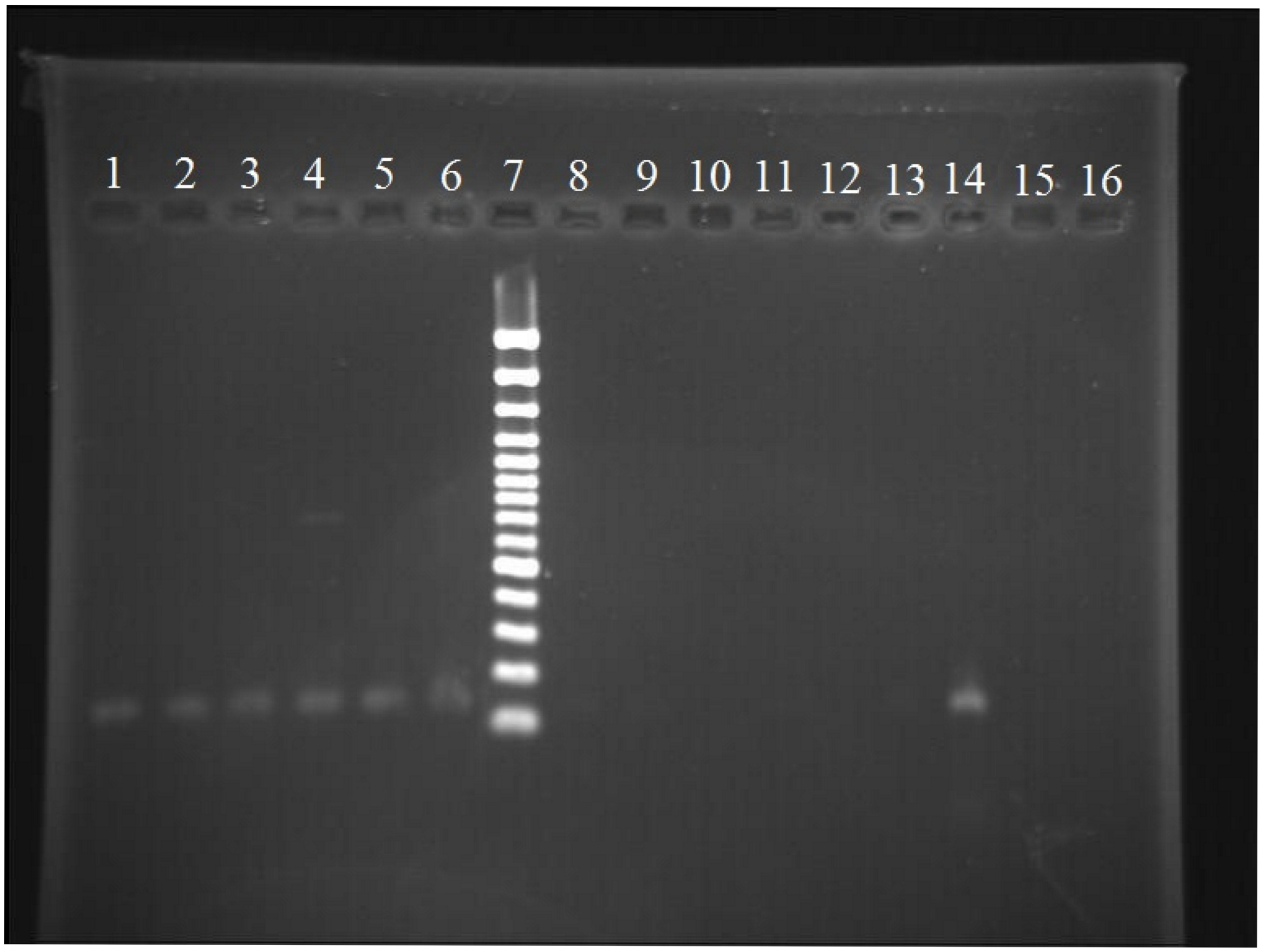
Agarose gel electrophoresis of *rrn*A-PCL1 for *Mycobacterium tuberculosis* (with band sizes 120 bp) From left to right: positive samples (1–6); molecular marker – 100 bp (7); negative samples (8–13); positive control (14); negative control (15–16)

Based on RT-PCR results, only 10 out of the 44 samples (23%) were positive for *M. tuberculosis* RNA. Of the 10 samples, 6 were related to the *rrn*A-PCL1 gene (Figure 1 (Fig. 1)) and 4 were related to the 16s rRNA gene. There was no significant difference between PCR and RT-PCR (p=0.403).

## Discussion

In our study, the prevalence of PTB and EPTB was 27% using PCR. To evaluate the metabolic activity of *M. tuberculosis*, the 16S rRNA and *rrn*A-PCL1 genes were examined using RT-PCR; the obtained frequency was 23%. This frequency rate is very close to that of *M. tuberculosis* DNA (27%) and confirms the presence of this bacterium in the suspected patients. However, the PCR method for detecting *M. tuberculosis* is more effective than the RT-PCR method. Although there was no statistically significant difference between PCR and RT-PCR, the PCR method in this study tended to be more effective (4.5%) than RT-PCR in detecting *M. tuberculosis* genes. RT-PCR can be used for the identification of *M. tuberculosis* with metabolic activity.

Our study suggests that in addition to culture, molecular methods should be used to assess the presence of latent TB. To assess the viability of *M. tuberculosis* in negative cultures, the detection of rRNA and mRNA of pathogenic bacteria is essential. The product of the *rrn*A-PCL1 gene has a significant increase in the log and stationary growth phases in *M. tuberculosis*. Therefore, one of the main components for verifying the metabolic activity of *M. tuberculosis* is the identification of the *rrn*A-PCL1 gene [11]. Some studies have investigated the frequency and prevalence of *M. tuberculosis* in clinical specimens. In a study conducted in Ahvaz (Iran) in 2012, the frequency of *M. tuberculosis* detected by PCR was 26%, while the frequency rate of *M. tuberculosis* detected in culture was 16% [12]. This study also demonstrates the sensitivity and specificity of molecular methods for the detection of *M. tuberculosis*. In another study conducted in 2008 in Tehran [13], the frequency of PTB and EPTB was examined by the purified protein derivative (PPD) skin test. In that study, of 262 patients with HIV and TB, 63 cases were postive according to PDD testing [13]. However, due to false positive results and very low specificity and sensitivity, PDD testing is not a suitable method for the detection of PTB and EPTB. In a study conducted in Taiwan in 2016 [14], the frequency of antibiotic-resistant *M. tuberculosis* was examined. That study was performed on cultured clinical samples collected from 2002 to 2014. The frequency of culture-positives was 7% in 2002 and about 9% in 2014 [14]. However, the culture method alone cannot be used to precisely determine the prevalence of *M. tuberculosis*. The prevalence of *M. tuberculosis* was investigated by Seyoum et al. in Ethiopia in 2014 [15]. In that study, 480 pulmonary specimens were examined. The frequency of *M. tuberculosis* was 12.5% [15], which is much lower than the TB frequency in our study. In addition, the frequency of *M. tuberculosis* was investigated in culture-negative pulmonary and extra-pulmonary specimens in Spain in 2012 [16]; that study found 13 positives and 53 negatives of 66 cultured clinical specimens. In this study, the IS*6110* gene was detected in 19 samples and rRNA in 35 samples; of 13 positive cultures, 8 were pulmonary samples and 5 were extra-pulmonary samples. In addition, the presence of the *rrn*A-PCL1 gene was investigated as an indicator for TB metabolic activity. The presence of the *rrn*A-PCL1 gene was confirmed in 11 samples [16]. This study demonstrates the importance of PCR for the detection of *M. tuberculosis*. It is known that the RT-PCR method, unlike PCR, is not a completely reliable method for the detection of *M. tuberculosis*. Furthermore, the sensitivity of the PCR method for the detection of *M. tuberculosis* was studied by Cheng et al. in 2008 [17], who found that out of 129 pulmonary and extra-pulmonary culture-positive samples, 10 were positive by PCR. The sensitivity of the PCR method was 78% [17].

The PCR method is a gold standard for the detection of *M. tuberculosis* [8]. In some cases, the sensitivity and specificity of this method for the detection of *M. tuberculosis* has been reported nearly 100%. Meanwhile, the specificity of the acid-fast staining and culture techniques for *M. tuberculosis* is about 87% [18]. Nagdev et al. reported the specificity of the PCR and RT-PCR methods for detecting *M. tuberculosis* in sputum specimens to be 92% and 84%, respectively [19]. According to the previous studies and the present study, the PCR method seems to be a reliable method for identifying *M. tuberculosis* in clinical samples. However, RT-PCR is also an appropriate method for the diagnosis of TB.

Altogether, management and control of TB (latent and active) is very important. Today, the evaluation of latent and active TB even in children is also important. A child with latent TB has a low number of *M. tuberculosis* bacteria, which usually yields a negative culture; however, this low number of bacterial cells is not destroyed by the immune system. The likelihood of the conversion of PTB from the latent to active form in adults is approximately 10%, while the possibility in children is about 40% [20].

## Conclusion

Considering the lack of an effective vaccine, high prevalence and antibiotic resistance of *M. tuberculosis*, control and treatment of this disease poses a global challenge. Today, new strategies are under development by the WHO for the control and treatment of TB. A 90% reduction in TB-related mortality and an 80% decline in TB incidence within 2030 are the main targets of this strategy [21].

It should be noted that AIDS is a major contributor to the increased TB mortality rate. About two-thirds of all TB cases worldwide are reported in Asia and Africa. AIDS, tobacco use, diabetes, and alcohol consumption are risk factors for the conversion of latent TB to active TB [21].

Given the progression of TB, assessing the prevalence and control of this disease should be prioritized. Investigations concerning the prevalence and treatment of latent TB should not be overlooked. According to previous studies, molecular methods are safe and effective for the detection of *M. tuberculosis* in suspected cases. Our study suggests that culturing is not a suitable method for identifying *M. tuberculosis* (especially the latent form). PCR is a highly sensitive and specific method for the diagnosis of TB. Overall, PCR is a reliable method to detect active and latent *M. tuberculosis* in comparison with RT-PCR.

## Notes

### Acknowledgment

The authors would like to thank the staff of Shahid Sadoughi hospital and Shahid Sadoughi University of Medical Sciences.

### Competing interests

The authors declare that they have no competing interests.

## Figures and Tables

**Table 1:**
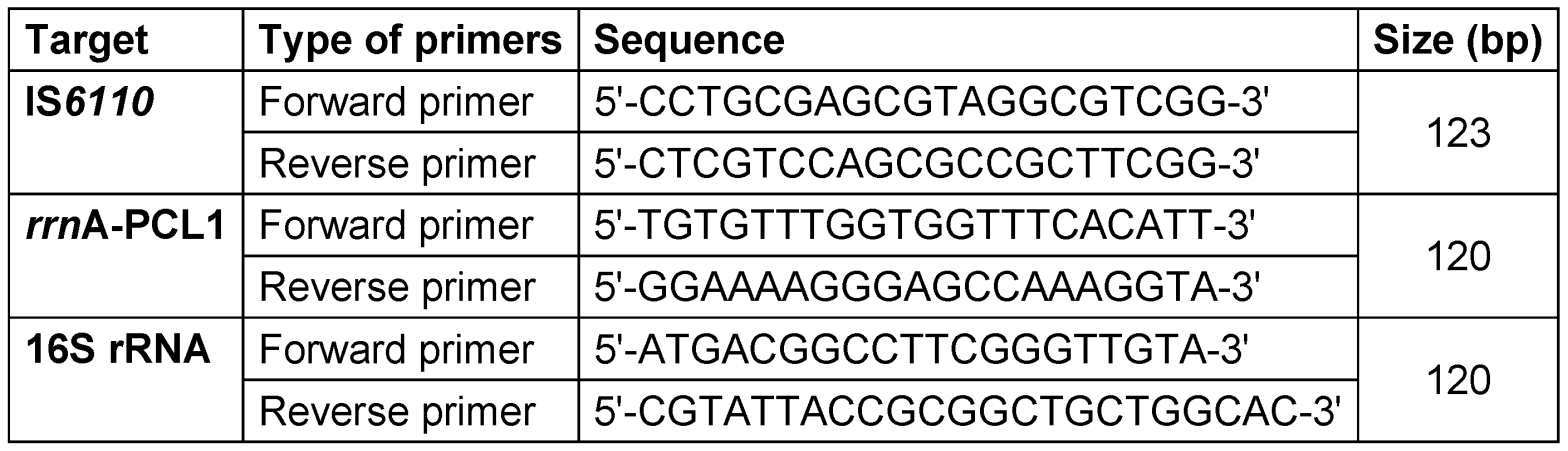
Primers for the detection of IS*6110* in PCR, *rrn*A-PCL1, and 16S rRNA in real-time PCR

**Table 2:**
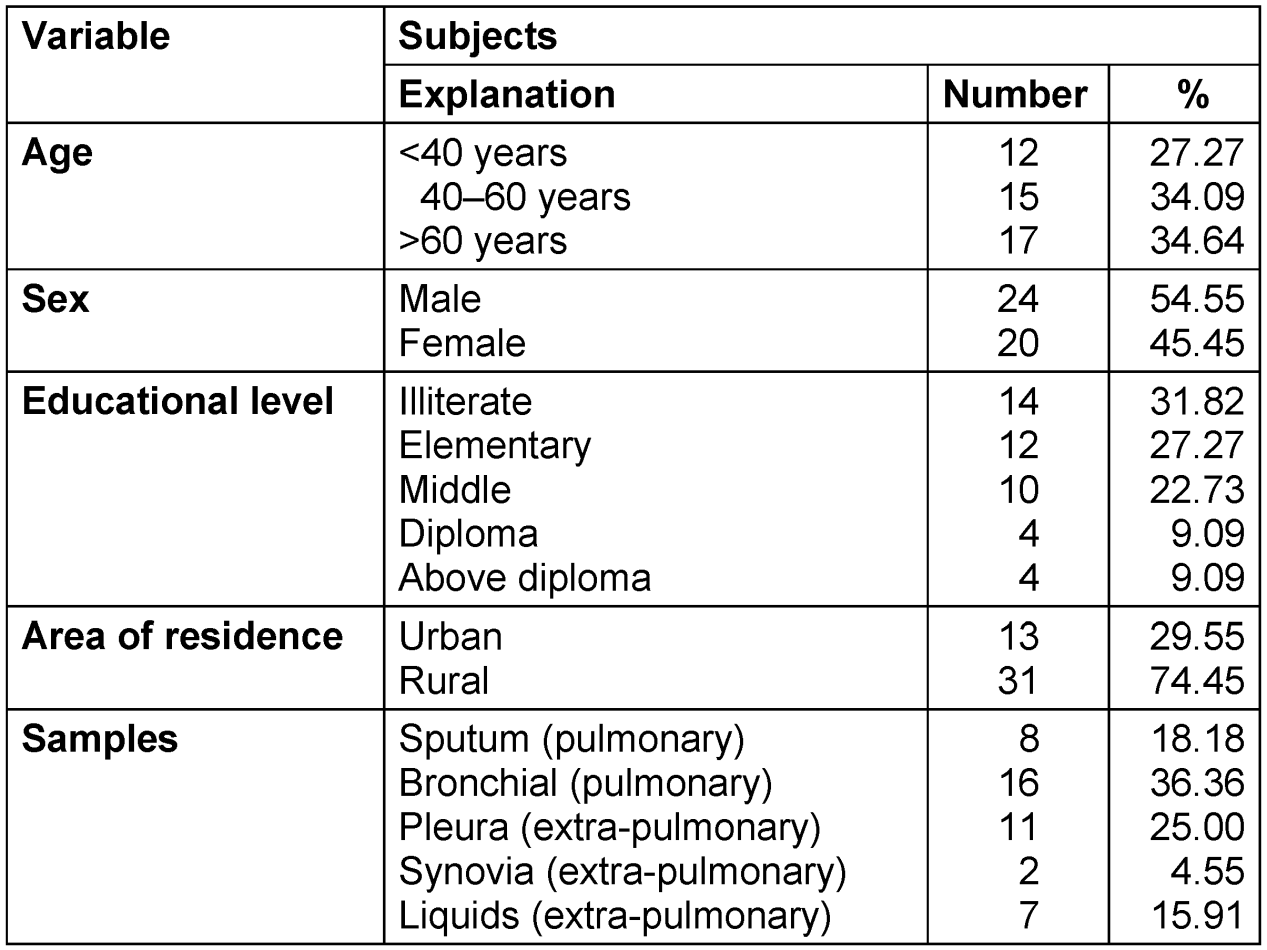
Demographic data of study subjects

**Table 3:**
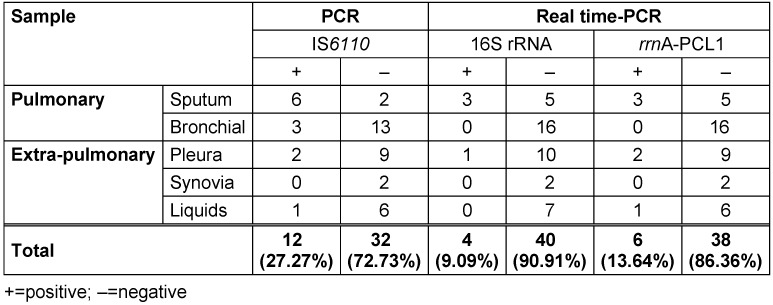
Results of culture and the identification of *Mycobacterium tuberculosis* nucleic acids in specimens
